# The Man Born Blind

**Published:** 1903-05-30

**Authors:** 


### "THE MAN BORN BLIND."

### The Emotional Side of the Halfpenny Press.

Under the first heading the *Daily Mail* has for about a week been giving a series of articles regarding a man who was operated upon in Glasgow for congenital cataract. Thus baldly stated, there does not seem much to write articles about. Congenital cataract is not very common, and this was justification enough for Dr. Maitland Ramsay, who performed the operation, giving an account of it in the pages of the *Lancet*. Dr. Ramsay's statement is simple, unpretentious, and interesting, and his remarks upon the effect of different colours upon the mind of the patient when he saw them for the first time, the extent to which the knowledge of form gained during his blindness helped him to recognise objects when he could use his eyes, and the way in which the gaining of sight destroyed that surety of movement which he had possessed when he was blind, are quite worth noting. Had, then, Dr. Ramsay's article been reproduced or quoted by the *Daily Mail*, no one could have complained. But that was not enough for our contemporary, which plunged into its characteristic emotionalism.

John Carruth, the "Man Born Blind," is a native of the village of Bridge-of-Weir. The place is described as "the most out-of-the-way part of an isolated village," where we are assured "they did not know of such places as the Ophthalmic Institution." Bridge-of-Weir is a village of about 2,500 inhabitants. As to its isolation, it is about seven miles from Paisley—a busy town where the sewing cotton comes from—and less than seven more from Glasgow, with which there is frequent railway communication. It is better known than many places of even greater size, because it is the site of the "Orphan Homes of Scotland," a large institution managed by Mr. William Quarrier, who is a Scottish Dr. Barnardo. Of late it has come into special prominence, as being the site chosen for a National Scottish Consumptive Sanatorium. Therefore it was not entirely unknown to fame, nor unvisited by the foot of the stranger, before John Carruth's eyes were operated upon. If Carruth and his parents did not know of the existence of an Ophthalmic Institution in Glasgow, they must have been exceptionally ill-informed people, dull beyond the average of Scottish peasants in districts far more remote. At least they knew of the Glasgow Asylum for the Blind, for Carruth has a sister there, and it is passing strange if they had never heard of an institution with which the asylum must have such intimate relations. That they may have thought that congenital blindness was beyond cure is likely enough, but not that they knew nothing of places where injured eyes were operated on. The operation is referred to as "a miracle of science." It is true indeed that when the miracles of science, like those of nature, become too familiar to us, we are apt to underrate the marvel of them. Perhaps we should indeed recognise an operation for cataract as a miracle. But oculists don't call it so now; something rarer is needed to arouse their wonder.

In short, the *Daily Mail* has striven to cast a spurious air of romance around a case which, although not common, is in no way specially remarkable. Had the reporter who interviewed Carruth been a keen psychologist, he might, even in such a case, have learned something worth telling the world. Dr. Ramsay noted something curious. Carruth took great pleasure in seeing anything red, but his first sight of yellow made him, he said, feel sick, though this was but a passing feeling, and did not recur. But the *Daily Mail* can only tell us that Carruth found the world beautiful, and women beautiful, and their dresses beautiful—though he thinks they wear a great many unnecessary adornments. He has been taken for a sail in a steamer on the Firth of Clyde, and found it beautiful. He has been taken to the Glasgow Zoo, and felt nervous in front of the tigers, and sympathetic towards the sweltering Polar bear. He has seen the King and Queen on their recent visit to Glasgow, and found their glistening cavalry escort wonderful. The whole thing is told as a sort of "Little Tommy's Holiday." There is nothing new in it, nothing that it imports the world to know. If Carruth could have expressed and the *Daily Mail* representative recorded the first impressions of a man who at the age of 30 beholds the world for the first time, they would indeed be well worth studying. Some such details on the physiological side Dr. Ramsay gives, but the *Daily Mail* gives nothing—nothing but oft-repeated platitudes.

We do not want to belittle the case. We who have enjoyed the blessing of sight all our lives are doubtless too much inclined to take it as a matter of course, but we can well understand how to Carruth and his parents the restoration of his sight is a marvel, a divine gift regarding which they cannot say enough. One would treat their delight and thankfulness with all reverence. And we can understand how the matter is a nine days' wonder to his neighbours. But the private joy is too sacred a thing to be exploited for the benefit of all the world, and on that side of the matter which the public might be supposed to have a right to be informed there is nothing to be said. An operation for a cataract of long standing has been successful; that is all. There is no legitimate material wherewith to fill, day after day, the columns of a daily newspaper belonging to a city 300 miles away. The title of the articles has evidently been chosen because of its scriptural flavour. And the *Daily Mail* might well have been content to sum up the matter in the simple Bible words, "Whereas I was blind, now I see."

